# Characterization of cyantraniliprole resistance in *Spodoptera frugiperda*: Selection, inheritance pattern, and cross‐resistance to other diamide insecticides

**DOI:** 10.1002/ps.8827

**Published:** 2025-04-17

**Authors:** Leonardo V Thiesen, Gabriela C Gonçalves, Aline S Guidolin, Antonio RB Nascimento, Everton F Coutinho, Jackeline P Borba, Eduardo CM Picelli, Celso Omoto

**Affiliations:** ^1^ Department of Entomology and Acarology Luiz de Queiroz College of Agriculture, University of São Paulo Piracicaba Brazil; ^2^ FMC Agricultural Solutions Paulínia Brazil

**Keywords:** fall armyworm, monitoring, I4790K mutation inheritance, cross‐resistance, insect resistance management

## Abstract

**BACKGROUND:**

Cyantraniliprole, a diamide insecticide, is widely used in Brazil to control sucking and defoliating pests, including the fall armyworm (*Spodoptera frugiperda*), a major agricultural pest. However, increasing resistance to diamides has raised concerns about the long‐term effectiveness of cyantraniliprole. This study aimed to (i) assess the evolution of cyantraniliprole resistance in field populations of *S. frugiperda*, (ii) investigate cross‐resistance to other diamides, and (iii) analyze the role of the I4790K mutation in resistance mechanisms.

**RESULTS:**

A significant decrease in the susceptibility to cyantraniliprole was observed in field populations of *S. frugiperda* in Brazil, particularly in regions with intensive agricultural systems, such as the Brazilian Cerrado, from 2017 to 2023. A cyantraniliprole‐resistant strain of *S. frugiperda* was selected from a field‐collected population in Bahia (BA) using the F_2_ screening method. The inheritance of resistance to cyantraniliprole in this strain was autosomal recessive and monogenic, with 3414‐fold resistance ratio. High cross‐resistance to the diamides flubendiamide, chlorantraniliprole, and cyclaniliprole was also detected. Molecular analysis confirmed the presence of the homozygous I4790K mutation in the ryanodine receptor gene as one of resistance mechanisms of this cyantraniliprole‐resistant strain.

**CONCLUSION:**

These findings highlight the decreased susceptibility of field populations of *S. frugiperda* to cyantraniliprole and the role of the I4790K mutation in accelerating the evolution of resistance to diamide insecticides due to cross‐resistance. These results underscore the urgent need for integrated pest management (IPM) strategies, including insecticide rotation and resistance monitoring, to preserve the efficacy of cyantraniliprole and other insecticides. © 2025 The Author(s). *Pest Management Science* published by John Wiley & Sons Ltd on behalf of Society of Chemical Industry.

## Graphical abstract

A cyantraniliprole‐resistant strain of *Spodoptera frugiperda* with I4790K mutation exhibited a resistance ratio of 3414‐fold and high cross‐resistance to other diamide insecticides.

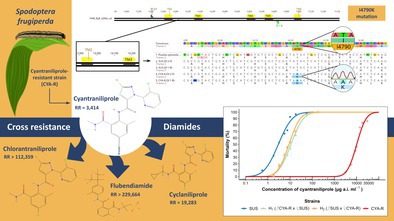

## 1 INTRODUCTION

Global food production is projected to increase by 36–56% by 2050,^1^, ^2^ and insect pests are a significant challenge in achieving this goal. The management of these pests has primarily relied on synthetic insecticides, specifically crops like maize and cotton, and genetically modified plants expressing Cry proteins from *Bacillus thuringiensis* (Bt). However, the overuse of insecticides has raised concerns regarding their environmental and human health impacts. Therefore, efforts are underway to develop selective insecticides that maintain efficacy while enhancing environmental and human safety.^3^

Among the newer insecticides, diamides stand out due to their selective action, low toxicity to non‐target organisms, and favorable environmental profile.^4^ These characteristics have contributed to diamides accounting for approximately 8% of the global insecticide market in recent years.^3^ A key marketing strategy for this group of compounds has been to promote their novel mode of action, which provides broad‐spectrum control over various pests, including both sucking and defoliating insects.^5^, ^6^, ^7^

Diamides are also widely used as seed treatments due to their high plant translocation and residual activity, providing essential early protection for crops when they are vulnerable to pest attacks.^8^, ^9^ The first two diamides, flubendiamide and chlorantraniliprole, were registered in 2007 to control lepidopteran pests.^6^ In 2013, new molecules such as cyantraniliprole were introduced, expanding the control spectrum while maintaining selectivity for non‐target species.^7^ Cyantraniliprole is particularly effective against sap‐feeding insects, including whiteflies, leafhoppers, aphids, psyllids, and lepidopterans, making it a valuable tool for integrated pest management programs.^4^, ^7^, ^10^

In Brazil, agricultural systems are characterized by extensive arable land and favorable climatic conditions, contributing to a significant increase in pest populations.^11^ These systems, primarily on soybean, maize, and cotton crops, can sustain up to three annual cultivation cycles in some regions, particularly in the Brazilian Cerrado, which has the largest arable area in the states of Bahia (BA), Mato Grosso (MT), Goiás (GO), Mato Grosso do Sul (MS) and Minas Gerais (MG).^12^ In Brazil, grain‐producing regions typically have three distinct crop seasons: the first‐season (October to January), the second‐season (February to May, marking the end of the rainy period), and the third/off‐season (June to September). This environment has particularly benefited polyphagous pests such as the fall armyworm, *Spodoptera frugiperda* (JE Smith, 1797) (Lepidoptera: Noctuidae), a globally significant pest and one of the main pests of maize crops, which has also been increasingly reported in cotton and soybean crops in Brazil.^13^, ^14^, ^15^

Continuous exposure of *S. frugiperda* to insecticides and Bt proteins has led to field‐evolved resistance to several insecticides, including avermectinas,^16^ benzoylureas,^17^, ^18^ spinosins,^19^, ^20^ organophosphates,^21^ diamides,^22^, ^23^ and Bt proteins.^24^, ^25^, ^26^, ^27^, ^28^ Resistance to diamides primarily occurs through mutations in the ryanodine receptor gene, which reduces the insecticide's binding affinity, leading to insensitivity.^29^, ^30^, ^31^ This resistance mechanism was first identified in *Plutella xylostella* (Lepidoptera: Plutellidae) in 2012, with mutations in the C‐terminal region of the transmembrane domain of the ryanodine receptor.^32^, ^33^ Subsequent studies have confirmed that alterations in specific amino acids, particularly G4946E, I4790M, and I4790K, are critical for conferring resistance to diamides. The I4790K mutation in *P. xylostella* has been associated with high levels of cross‐resistance among all diamides, affecting both anthranilic diamides and those derived from phthalic acid.^29^, ^34^ Recent reports indicate that both I4790M and I4790K mutations are present in Brazilian populations of *S. frugiperda*.^23^ However, the role of the I4790K mutation in homozygous individuals remains unclear, as only a few individuals with the I/K genotype have been documented to date.

This study aimed to evaluate the changes in the susceptibility of fall armyworm field populations to cyantraniliprole over different crop seasons from 2017 to 2023. Furthermore, we aimed to select and characterize an *S. frugiperda* strain resistant to cyantraniliprole to understand the inheritance of resistance and cross‐resistance to other diamide insecticides. The findings from this study will contribute to understanding the evolution of *S. frugiperda* to cyantraniliprole and implement strategies to manage this resistance.

## 2 MATERIALS AND METHODS

### 2.1 Insects

The *S. frugiperda* susceptible strain (SUS) used in the bioassays was reared on an artificial diet^35^ without exposure to insecticides or Bt toxins for successive generations under laboratory conditions. The strain resistant to the insecticide cyantraniliprole (CYA‐R) was selected from surviving individuals using the F_2_ screen method^36^ from a population collected in Luis Eduardo Magalhães, Bahia, Brazil (11°44′48.90″ S, 45°46′07.60″ W). After selection, a concentration‐response curve was estimated in the F_3_ generation CYA‐R to determine the highest concentration for the selection pressure to select homozygous individuals (~10% of survival). From the F_4_ generation, CYA‐R was maintained at a constant selection pressure at a cyantraniliprole concentration of 3200 μg a.i. mL^−1^.

For susceptibility monitoring of *S. frugiperda* to cyantraniliprole, 1000–1500 larvae were initially collected from field populations in major maize‐producing regions of Brazil from 2017 to 2023 crop years (Table S1). After collecting, a screening process was conducted to ensure the viability and condition of the larvae. Following this triage, the viable larvae were transported to our laboratory, where they were reared on an artificial diet until they reached the pupal stage. After disinfection with 6.5% copper sulfate solution, pupae were placed in cylindrical PVC cages (10 cm diameter × 20 cm height) lined with white paper and closed at the top with plastic containers. Adults were fed a 1:10 honey: water solution. Egg masses were collected every 2 days and placed in plastic cups (100 mL) until the larvae hatched. Neonate larvae were transferred to new plastic cups containing an artificial diet, where they remained until the third instar, when they were used in the bioassays. All developmental stages were maintained in a climate‐controlled room (25 ± 2 °C, 60 ± 10% relative humidity, and a 14:10 h [L:D] photoperiod).

### 2.2 Monitoring the susceptibility of *Spodoptera frugiperda* to cyantraniliprole

A diet‐overlay bioassay method was used to monitor the susceptibility of *S. frugiperda* to cyantraniliprole (Benevia® OD, 100 g a.i. l^−1^, FMC Química do Brasil LTDA, Campinas, SP, Brazil). Bioassays were performed in 24‐cell acrylic plates (1.9 cm^2^ in area) (Costar® Maizeing Incorporated, Sigma‐Aldrich Brazil Ltd., São Paulo, BR), containing 1.25 mL of artificial diet per well, where 30 μL of insecticide solution and surfactant (Triton® X‐100, Labsynth, Diadema, SP, Brazil) at a concentration of 0.1% (v/v) were pipetted to each well. The diagnostic concentration was 180 μg a.i. mL^−1^ ^37^ and after drying the insecticide solution, 480 third‐instar larvae were infested with each field population (one larva/well), totaling 20 replicates of 24 larvae. The plates were incubated in a climate‐controlled chamber (25 ± 2 °C, 60 ± 10% relative humidity, 14:10 h [L:D] photoperiod), and mortality was assessed 96 h after exposure. Larvae that failed to move normally when touched with a brush were considered dead.

A meta‐analysis was conducted to evaluate the survival of *S*. *frugiperda* exposed to cyantraniliprole, utilizing the survival data collected during our study (2017–2023). This analysis also incorporated data available in the literature regarding the efficacy of other two diamide insecticides tested in Brazil.^37^, ^38^, ^39^ For this meta‐analysis, the survival data to cyantraniliprole were analyzed using the non‐parametric Kruskal–Wallis test,^40^ followed by mean comparisons using Dunn's test^41^ with Bonferroni correction. The susceptibility of field populations of *S. frugiperda* to cyantraniliprole, chlorantraniliprole, and flubendiamide was assessed through historical data monitoring from literature^37^, ^38^, ^39^ and our study. A generalized linear mixed model (GLMM) was fitted using Template Model Builder, implemented in the ‘glmmTMB’ package^42^ This model assumed a binomial distribution using smoothing terms within GAM model formulae, with insecticides and the States of origin of the populations as random variables. Graphical analysis was performed using predicted values from the GLMM, employing the ‘loess’ method to construct regressions for the predicted survival response variable. All analyses and visualizations were conducted in R software version 4.3.1.^43^

### 2.3 F_2_ screen to estimate the resistance alleles frequency

To estimate the frequency of resistance alleles using the F_2_ screen method^36^ we evaluated seven populations collected in regions with intensive maize planting systems in Brazil (up to three crop seasons per agricultural year) between the 2022 and 2023 crop years (Table 1). In the pupal stage, individuals were separated by sex, and approximately 190 couples were formed and considered isolines. Each couple was maintained in 500 mL plastic cups containing a 1:10 honey: water solution, and egg masses were collected every 2 days. After the F_1_ progeny reached the third instar, they were transferred to 32‐cell plates containing an artificial diet. Three replicates per isoline (96 larvae) were reared until pupation.

**Table 1:** Location and number of individuals (n) of *Spodoptera frugiperda* after screening from field populations for monitoring using the F_2_ screen method

Code	Location (City, State)	*n* ^†^	Crop Year^‡^	Arrived date	Latitude (S)	Longitude (W)
GO‐19	Cristalina, GO	1168	2022/1	27 October 2021	16°29′59.80″	47°36′36.80″
BA‐9	Luís Eduardo Magalhães, BA	1400	2022/1	11 March 2021	11°44′48.90″	45°46′07.60″
MT‐20	Lucas do Rio Verde, MT	1100	2022/2	22 March 2022	13°01′38.30″	55°57′11.40″
MS‐14	Chapadão do Sul, MS	730	2022/2	19 April 2022	18°44′23.54″	52°31′03.02″
C_BA‐6 (cotton)	Luís Eduardo Magalhães, BA	894	2022/3	31 May 2022	12°01′41.84″	45°45′02.32″
GO‐22	Santa Helena, GO	830	2023/1	26 October 2022	17°51′37.09″	50°23′34.07″
BA‐10	São Desidério, BA	1226	2023/1	14 December 2022	13°40′15.32″	46°07′37.54″

^†^ Number of insects tested.

^‡^ Crop year started in October of the previous year and ended in September of the following year, split into approximately three seasons of 4 months each.

The same procedures were used for breeding and genotyping the F_2_ generation. Cages containing the F_1_ adult progeny of each isoline were maintained to collect eggs from the F_2_ generation for monitoring. The bioassay was a diet overlay in which the diet was treated with cyantraniliprole at a discriminatory concentration of 180 μg a.i. mL^−1^. For each isoline, approximately 120 third‐instar larvae were tested, and isolines that showed survival greater than 6.25% were considered positive, based on the premise that 1 out of every 16 individuals derived from a susceptible and heterozygous parent collected from the field would exhibit homozygosity for the resistance allele in F_2_ generation. Resistance allele frequency was estimated according to the formula proposed by Andow and Alstad^36^:

(1) $$q=\left(S+1\right)/\left(\left(2+N\right)\times 4\right)$$

where *q* is the estimated allele frequency of the resistance, *S* is the number of positive isolines, and *N* is the number of tested isolines.

The frequencies estimated by the F_2_ screen were utilized to develop predictive models based on available literature data for monitoring the frequency of resistance alleles to diamides in *S. frugiperda* in Brazil.^37^, ^38^, ^39^ Using this database of allele frequency estimates for diamides and field populations of FAW from 2012 to 2022, two predictive models were highlighted to demonstrate the trends in frequency increase: one for the Bahia region (worst scenario) and another for all areas within the Brazilian Cerrado. To explore resistance evolution trends, we applied a GLMM incorporating crop season as a fixed effect and annual variability as a random effect, with smoothing terms following a GAM model structure.^42^ All analyses and visualizations were performed using R software version 4.3.1.^43^

### 2.4 Inheritance of resistance of *Spodoptera frugiperda* to cyantraniliprole

Diet‐overlay bioassays were performed after 10 generations of the CYA‐R strain in the laboratory to characterize the genetic basis of the resistance. The pupae from SUS and CYA‐R strains were separated by sex and kept in plastic cups (50 mL). As the adults emerged, reciprocal crosses between 25 couples (♀CYA‐R × ♂SUS and ♀SUS × ♂CYA‐R) were kept in cages (PVC tubes 10 cm in diameter × 20 cm in height). The heterozygous progenies of the reciprocal crosses (F_1_) were subjected to diet‐overlay bioassays to perform concentration‐response curves for cyantraniliprole and estimate the LC_50_ for each heterozygous strain using logarithmic concentrations spaced between 1.80 and 180 μg a.i. mL^−1^ of cyantraniliprole.

The possibility of resistance being linked to sex or maternal effects was verified by analyzing the results obtained in reciprocal crossings. The average degree of resistance dominance was estimated by comparing the LC_50_ of the F_1_ progenies of the heterozygous lines with the LC_50_ of the parental strains using the formula proposed by Stone^44^:

(2) $$D=\left(2X_{\mathrm{RS}}-X_{\mathrm{RR}}-2X_{\mathrm{SS}}\right)/\left(X_{\mathrm{RR}}-X_{\mathrm{SS}}\right)$$

where *D* is the average degree of dominance, and *X*_*SS*_, *X*_*RR*_, and *X*_*RS*_ = log_10_ (LC_50_) of the SUS strain, CYA‐R, and H_1_ and H_2_ heterozygotes, respectively. The value of D varies between −1 and 1, with resistance being completely dominant when D = 1, incompletely dominant when 0 < D < 1, incompletely recessive when −1 < D < 0, and completely recessive when D = −1. Complementary to the method proposed by Stone,^44^ mortality data from bioassays were submitted to the formula proposed by Bourguet *et al*.^45^ to verify the degree of dominance at different concentrations:

(3) $$D=\left(M_{\mathrm{RS}}-M_{\mathrm{SS}}\right)/\left(M_{\mathrm{RR}}-M_{\mathrm{SS}}\right)$$

where *D* is the average degree of dominance, and *M*_*SS*_, *M*_*RR*_, and *M*_*RS*_ are the mortality of the SUS, CYA‐R, and heterozygous strains, respectively. D values close to zero indicate completely recessive inheritance, whereas values close to one indicate completely dominant inheritance.

The inheritance was analyzed for its monogenic nature using the 𝜒^2^ test between observed and expected mortality data from backcrosses.^46^ The expected mortality from backcrossing at a given concentration was calculated based on the average mortality between the F_1_ progeny and their parents proposed by Sokal and Rohlf^47^

(4) $$\chi^{2}={\left(\mathrm{Ni}–\left(p\times \mathrm{ni}\right)\right)}^{2}/\left(p\times q\times \mathrm{ni}\right)$$

where *Ni* = Mortality observed from backcrossing of progeny to concentration *i, p* is the proportion of mortality calculated by the Mendelian model (p = (*a* + *b*) / 2), *ni* is the number of progeny individuals that were tested at concentration *i, q* is the proportion of expected survival mortality (*q* = 1 – *p*), *a* is the mortality of the parental strain, and *b* is the mortality of the heterozygous progeny F_1_. The monogenic inheritance hypothesis was rejected when χ^2^_calculated_ ≥ χ^2^_tabulated_ with degrees of freedom equal to 1 (*P* < 0.05).

To estimate the LC_50_, a generalized linear model (GLM) was utilized, assuming a binomial distribution and a probit link function. The quality of the model fit was assessed using the χ^2^ test and a ‘half‐normal plot’ with simulated envelopes from the hnp package.^48^ The LC_50_ value was estimated using the ‘dose.p’ function from the MASS package,^49^ and the heterozygous strains were compared using the parallelism and equality tests by comparing models with complete interactions and reduced models using the Chi‐square test. All analyses and visualizations were performed using R software version 4.3.1.^43^

### 2.5 Cross‐resistance between cyantraniliprole and other diamide insecticides

To verify the cross‐resistance of *S. frugiperda* resistant to cyantraniliprole and flubendiamide (Belt®, SC 480 g a.i. l^−1^, Bayer SA, São Paulo/SP, Brazil), chlorantraniliprole (Premio®, SC 200 g a.i. l^−1^, FMC Quimica do Brasil Ltda., Campinas/SP, Brazil), and cyclaniliprole (Goemon®, SL 50 g a.i. l^−1^, ISK Biosciences do Brasil, Indaiatuba/SP, Brazil), concentration‐response curves were obtained for each insecticide for the SUS and CYA‐R strains. The bioassay method was the same as that described previously, and mortality was assessed 96 h after exposure. The presence or absence of cross‐resistance was verified by comparing the resistance ratio (RR) by dividing the LC_50_ of the CYA‐R strain by the LC_50_ of the SUS strain for each insecticide.

### 2.6 Molecular and genotypic analysis of the ryanodine receptor in cyantraniliprole‐resistant *Spodoptera frugiperda*

To investigate the molecular basis of cyantraniliprole resistance in *S*. *frugiperda*, total RNA was extracted from 30 early third‐instar larvae of both SUS and CYA‐R strains using the RNeasy® Plant Mini Kit (Qiagen). RNA samples were treated with DNase I to remove any contaminating genomic DNA (Thermo Scientific, Waltham, MA, USA), following the manufacturer's protocol. RNA quantity and integrity were assessed using a NanoDrop spectrophotometer (Thermo Scientific, Waltham, MA, USA) and 1.0% agarose gel electrophoresis. RNA was normalized to 1000 ng.μl^−1^, and 1 μg was used to synthesize first‐strand cDNA using the SuperScript® III First‐Strand Synthesis System for RT‐PCR (Invitrogen) and Oligo(dT)20 primer (Promega, Madison, WI, USA).

To sequence the ryanodine receptor (RyR), primers targeting transmembrane domains II to V were designed (Table 2) based on the C‐terminal region of the RyR gene.^50^ The primers amplified two fragments of 740 and 1038 base pairs (bp). PCR amplification was performed using Q5 High‐Fidelity DNA polymerase (New England BioLabs). Each 50 μL PCR reaction contained 10 μL of buffer, 1 μL of dNTPs, 0.5 μL of Q5 DNA polymerase, 2.5 μL of each primer, and 5 μL of 10x diluted cDNA, with MilliQ water added to reach the final volume. The PCR conditions were 35 cycles at 98 °C for 10 s, 60 °C for 30 s, and 72 °C for 30 s, followed by a final extension at 72 °C for 2 min. The PCR products were analyzed using 1% agarose gel electrophoresis, purified using Beckman Coulter™ Agencourt AMPure XP magnetic beads (Beckman Coulter, Inc. Brea CA 92821, U.S.A), and subsequently sent for Sanger sequencing. The Sanger sequencing results were aligned with the *P. xylostella* ryanodine receptor reference sequence (NP_001296002) to identify potential mutations.

**Table 2:** Primers used to amplify the region covering transmembrane domains II to V of *S. frugiperda* ryanodine receptors

Primer	Sequences (5′ → 3′)
*Sf 1‐F*	ACGACGATGCACTAGAAG
*Sf 2‐F*	GCCATCGAAGCTGAGAGCAA
*Sf 1‐R*	GTTCCTGTTGACCTCGTCGT

Individual genotyping was performed on 30 third‐instar larvae from both the SUS and CYA‐R strains to verify the presence of I4790K mutation in the ryanodine receptor. Genomic DNA (gDNA) was extracted using Chelex 100 Resin (Bio‐Rad, Hercules, CA, USA) at 10%, normalized, and used in real‐time quantitative PCR (RT‐qPCR) assays. The reactions were conducted on a QuantStudio™ 5 System (Thermo Scientific, Waltham, MA, USA) using the TaqPath™ ProAmp Master Mix kit. Each 20 μL qPCR reaction contained 5 μL of 3 ng.μl^−1^ gDNA, 10 μL of TaqPath™ ProAmp Master Mix, 10 μM of each primer, 0.4 μL of mutation‐specific probes, and MilliQ water. The qPCR protocol involved an initial denaturation step at 95 °C for 5 min, followed by 40 cycles at 95 °C for 15 s and 60 °C for 30 s. Primers and probes for detecting the I4790K mutation were designed as described by Boaventura *et al*.^50^ and Okuma *et al*.^23^ (Table 3).

**Table 3:** Primers and probes used for genotyping *S. frugiperda* strains by RT‐qPCR

Sequences (5′ → 3′)	Orientation	Modifiers	References
CGACGATGCACTAGAAGTG	Forward	–	Mod. from Boaventura *et al*.^50^
ACCTTGAGATGGTAGTACC	Reverse	–	Mod. from Boaventura *et al*.^50^
TGCTCGCTAAACTCATCGGGT	Probe	5’ FAM + 3’ BHQ1	*Sf*. I4790K mod. from Okuma *et al*.^23^
TGTCGCTCGCTATACTCATCG	Probe	5’ HEX +3’ BHQ1	*Sf*. I4790I mod. from Boaventura *et al*.^50^

## 3 RESULTS

### 3.1 Monitoring the susceptibility of *Spodoptera frugiperda* to cyantraniliprole

**Figure 1:**
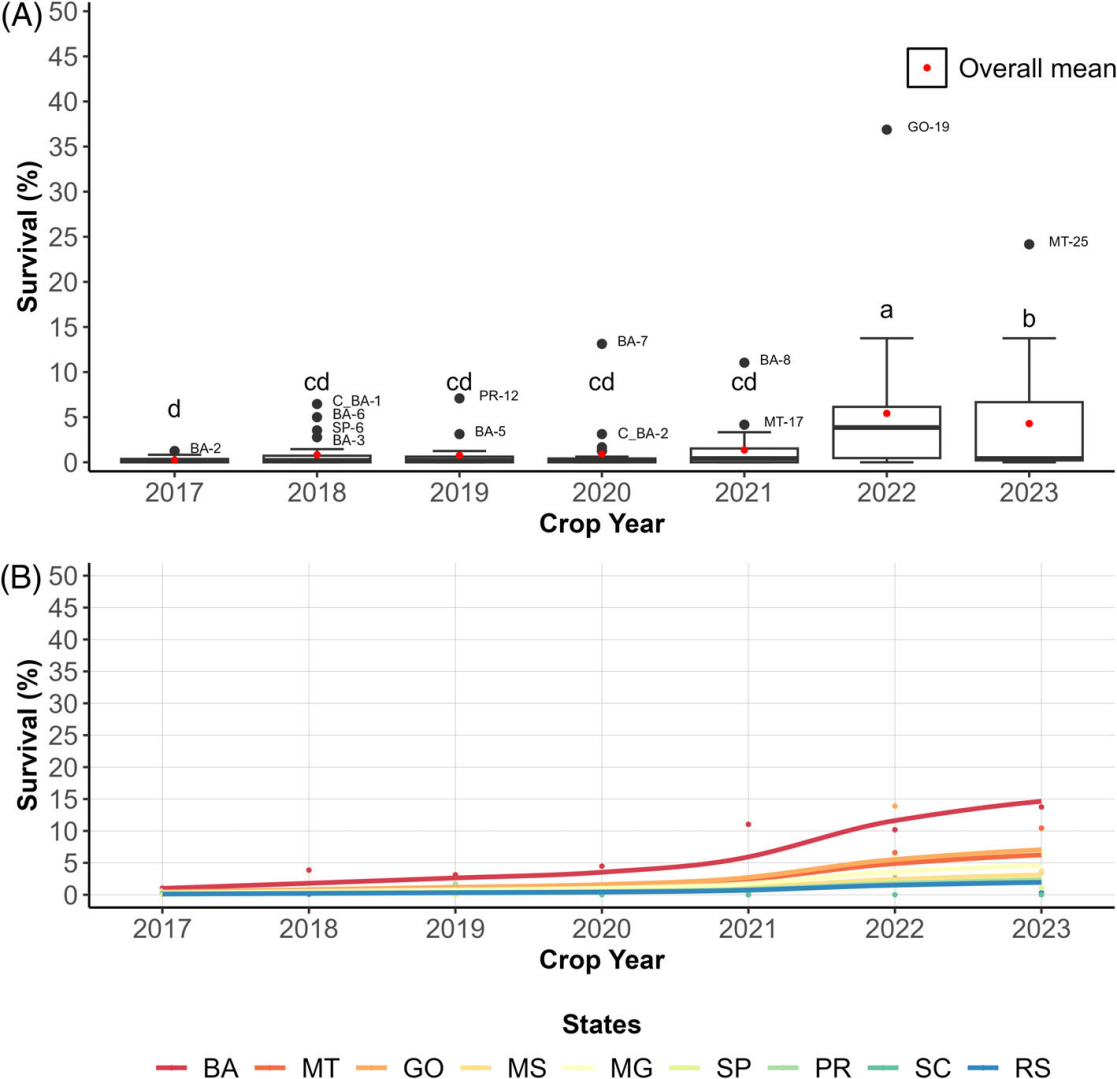
Average survival (%) of field populations of *S. frugiperda* monitored for the insecticide cyantraniliprole. (A) Average variation in survival over the crop years. Different letters in each box indicate differences between years of average survival, according to Dunn's test (*P* < 0.05). (B) Predicted GLMM of the survival behavior of *S. frugiperda* populations separated by state.

The average survival rate of field populations of *S*. *frugiperda* has remained below 10% since laboratory monitoring began in 2017. However, outliers were observed, with survival rates between 12.39% and 14.03% in specific regions of Goiás (GO) and Bahia (BA) (Fig. 1(A)). The average survival rate in the 2022 crop season was significantly higher (3.88% ± 4.35) than in the previous year (2.03% ± 4.41), showing considerable variation across different locations. The generalized linear mixed model (GLMM) revealed that survival rates in BA were higher, followed by Mato Grosso (MT) and GO (Fig. 1(B)) (Intercept = 0.0391 and β = 0.0514; *P* = 0.0348).

### 3.2 F_2_ screen to estimate the resistance allele frequency

The estimated frequency of the resistance allele for cyantraniliprole was determined in seven field populations of *S. frugiperda*, with 75 644 larvae assessed at a discriminatory concentration. The highest estimated frequency of the resistance allele was observed in western BA, where it remained constant throughout the 2022–2023 crop season, with an average frequency of 0.1442. This suggests the persistence of resistant individuals in the region (Table 4). In the states of GO and MT, the estimated average frequencies were 0.0756 and 0.0734, respectively, representing a proportion of resistant individuals (Wrr) almost four times lower than in BA. The least critical scenario was observed in Mato Grosso do Sul (MS), where the estimated frequency was 0.0408 (Table 4).

**Table 4:** Frequency of resistance alleles to cyantraniliprole in *S. frugiperda* populations in Brazil

Code	Initial isolines	Tested isolines	Tested insects	Positive isolines	Frequency (q) (CI 95%)
GO‐19	190	123	14.107	50	0.1020 (0.0770–0.1299)
GO‐22	189	103	12 816	18	0.0453 (0.0275–0.0671)
**Goiás (GO)**	**379**	**226**	**26 923**	**68**	**0.0756 (0.0594–0.0936)**
BA‐9	186	69	8033	36	0.1303 (0.0937–0.1717)
C_BA‐6	154	48	5760	34	0.1750 (0.1257–0.2305)
BA‐10	201	115	13 568	64	0.1389 (0.1091–0.1716)
**Bahia (BA)**	**541**	**232**	**27 361**	**134**	**0.1442 (0.1224–0.1674)**
MT‐20	176	90	10 800	26	0.0734 (0.0490–0.1021)
MS‐14	184	90	10 560	14	0.0408 (0.0230–0.0632)
**Cerrado (all)**	**1280**	**638**	**75 644**	**243**	**0.0949 (0.0838–0.1065)**

### 3.3 Inheritance of resistance of *Spodoptera frugiperda* to cyantraniliprole

**Figure 2:**
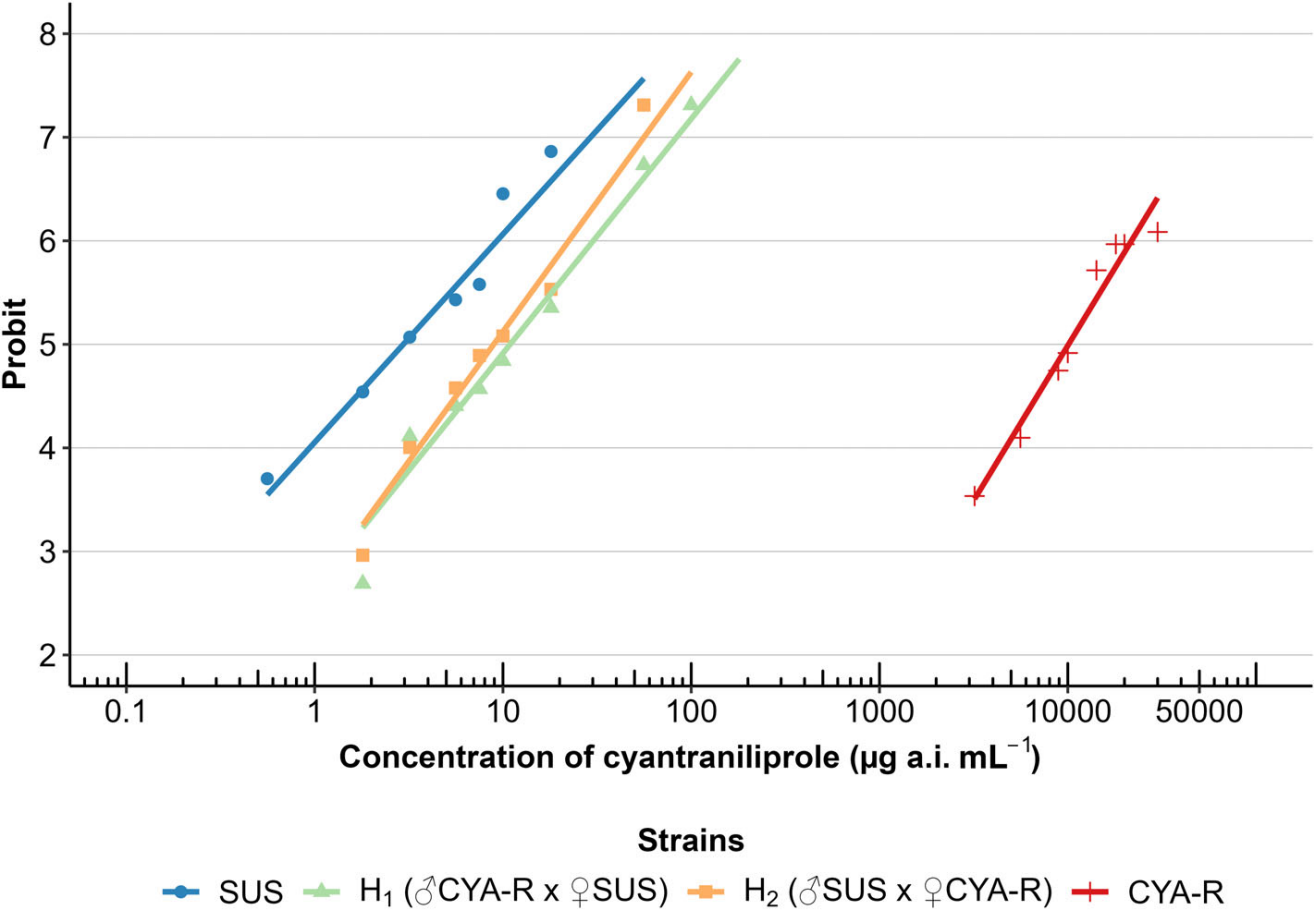
Probit lines for different strains of *S. frugiperda* exposed to the insecticide cyantraniliprole.

**Figure 3:**
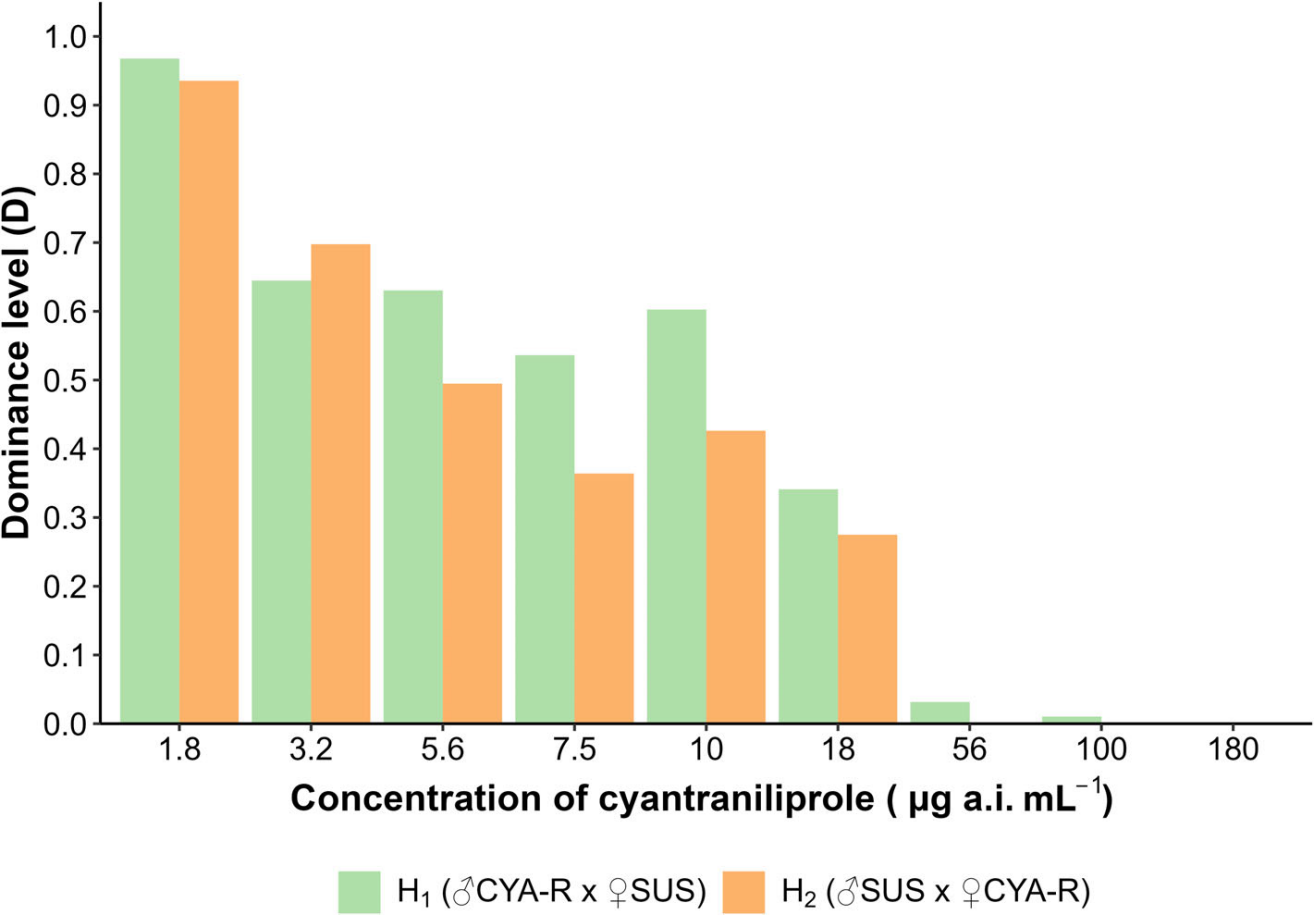
Degree of dominance of heterozygous strains of *S. frugiperda* as a function of different concentrations of cyantraniliprole using the formula proposed by Bourguet *et al*. (2000).^45^

The resistant strain selected by the F_2_ screen method (CYA‐R) showed an LC_50_ estimated at 10095.31 μg a.i. of cyantraniliprole mL^−1^, exhibiting a resistance ratio of 3414‐fold (Table 5). Both heterozygotes showed slight variations in the estimated LC_50_ values, yielding resistance ratios of 3.69‐fold for H_1_ (♂CYA‐R × ♀SUS) and 3.02‐fold for H_2_ (♂SUS × ♀CYA‐R) (Table 5). These findings suggest that inheritance is likely autosomal, as the heterozygotes exhibited resistance ratios that were qualitatively similar between the two strains based on the parallelism test (χ^2^ = 1.22, df = 1, *P* = 0.27), although not equal (χ^2^ = 6.43, df = 2, *P* = 0.04). Moreover, heterozygotes showed resistance levels closer to those of the SUS strain, as reflected by the low resistance ratio, indicating a recessive inheritance pattern (Fig. 2). This result was further supported by the negative values of the dominance degree (D) calculated using Stone's formula^44^ (Table 5) (Fig. 3). At diagnostic concentrations (180 μg a.i. of cyantraniliprole mL^−1^), the results of dominance indicated that the inheritance pattern is completely recessive (Fig. 3).

**Table 5:** Concentration‐mortality responses of *S. frugiperda* strains susceptible, heterozygous, and resistant to cyantraniliprole

Strains	*n* ^†^	LC_50_ ^‡^ (μg a.i. mL^−1^) (CI 95%)	χ^2^ (df)^§^	*p*	Slope (± SE)	RR_50_ (CI 95%)^‖^	D^¶^
SUS	804	2.96 (2.56–3.42)	10.24 (6)	0.12	2.01 (± 0.14)	–	–
H_1_ (♂CYA‐R × ♀ SUS)	907	10.92 (9.67–12.33)	9.60 (7)	0.21	2.26 (± 0.14)	3.69 (3.07–4.44)	−0.68
H_2_ (♂SUS × ♀ CYA‐R)	941	8.93 (8.00–9.96)	6.30 (7)	0.51	2.51 (± 0.17)	3.02 (2.49–3.66)	−0.73
CYA‐R (F_11_)	683	10 095.31 (9216.24–11 058.23)	8.33 (6)	0.21	2.99 (± 0.20)	3414.29 (2808–4141)	–

^†^ Number of insects tested.

^‡^ Lethal concentration of the applied insecticide solution that kills 50% of individuals, with a 95% confidence interval.

^§^ Chi‐square test (degrees of freedom).

^**‖**^ Resistance ratio (RR) obtained by dividing the LC_50_ of heterozygous (H_1_/H_2_) and resistant (CYA‐R) strains by the LC_50_ of the susceptible strain (SUS).

^¶^ Degree of dominance obtained using Stone's formula (1968).^44^

The hypothesis of monogenic inheritance was accepted by comparing the observed mortality results with the mortality data expected from the Mendelian model using the chi‐square test (Table 6).

**Table 6:** Chi‐square test to compare the observed and expected mortality (%) of backcrosses between the heterozygous strain (F_1_) and the cyantraniliprole‐resistant strain (CYA‐R) of *S. frugiperda*

Concentration	♂ F_1_ × ♀ CYA‐R				♂ CYA‐R × ♀ F_1_			
(μg. a.i. mL^−1^)	Obs^†^	Exp^‡^	χ^2^ ^§^	*p* ^**‖**^	Obs	Exp	χ^2^	*P*
18.00	40	33	0.82	0.37	33	33	0.05	0.82
56.00	41	49	2.50	0.11	51	49	0.24	0.63
100.00	52	50	0.21	0.65	50	50	0.00	1.00
180.00	53	50	0.42	0.52	53	50	0.53	0.47

^†^ Observed mortality (%).

^‡^ Expected mortality (%) according to Mendelian monogenic inheritance.

^§^ Chi‐square results.

^**‖**^ Significance level of 5% probability (χ^2^ >3.84, df = 1, *P* < 0.05).

### 3.4 Cross‐resistance between cyantraniliprole and other diamide insecticides

Cross‐resistance was confirmed between cyantraniliprole and all the other diamides tested. Our results showed that for the two first commercialized diamides, flubendiamide and chlorantraniliprole, the resistance ratios were >229 664‐fold and 112 359‐fold, respectively, indicating that even undiluted commercial formulations were insufficient to achieve 50% mortality. The resistance ratio for cyclaniliprole was also significant, at 19283‐fold, demonstrating that the strain resistant to cyantraniliprole exhibited high cross‐resistance to all diamides tested in Brazil (Table 7).

**Table 7:** Concentration‐mortality responses of cyantraniliprole‐susceptible (SUS) and cyantraniliprole‐resistant (CYA‐R) *Spodoptera frugiperda* strains to different diamides

Insecticide	Strains	*n* ^†^	LC_50_ ^‡^ (μg a.i. mL^−1^) (CI 95%)	χ^2^ (df)^§^	*P*	Slope (± SE)	RR_50_ ^‖^
Flubendiamide	SUS	600	2.09 (1.67–2.61)	6.21 (5)	0.28	1.25 (± 0.11)	–
	CYA‐R	624	>480 000.00	–	–	–	>229 664.00
Chlorantraniliprole	SUS	792	1.78 (1.51–2.11)	6.13 (6)	0.41	1.67 (± 0.11)	–
	CYA‐R	827	>200 000.00	–	–	–	>112 359.00
Cyclaniliprole	SUS	669	0.12 (0.10–0.15)	1.38 (6)	0.96	1.52 (± 0.10)	–
	CYA‐R	876	2488.55 (2199.16 – 2816.01)	11.67 (9)	0.23	2.10 (± 0.12)	19 283.67

^†^ Number of insects tested.

^‡^ Lethal concentration of the applied insecticide solution that kills 50% of individuals, with a 95% confidence interval.

^§^ Chi‐square test (degrees of freedom).

^**‖**^ Resistance ratio (RR) obtained by dividing the LC_50_ of the resistant strain (CYA‐R) by the LC_50_ of the susceptible strain (SUS).

### 3.5 Prediction of resistance evolution of diamides insecticides to *S. frugiperda*

**Figure 4:**
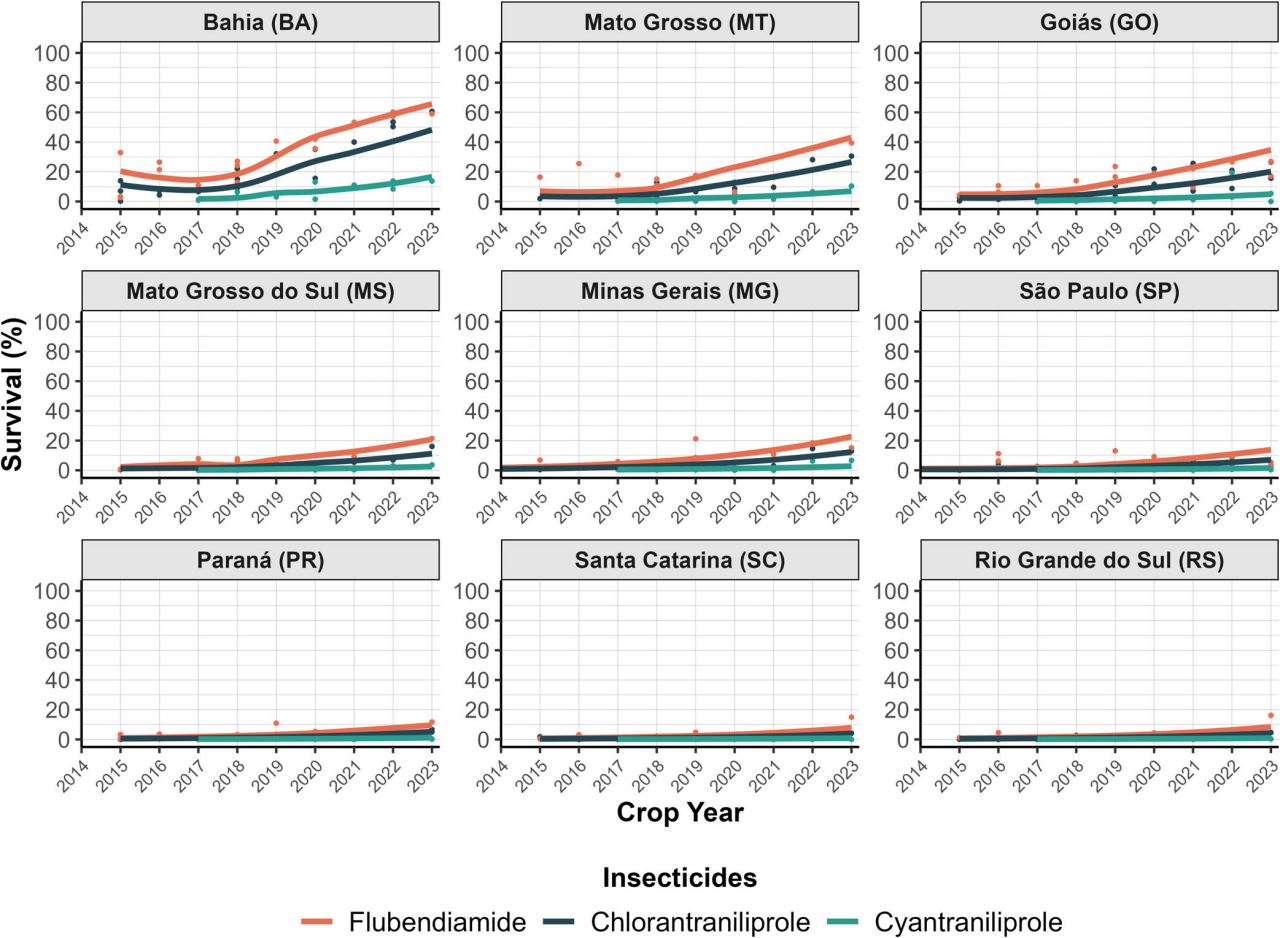
Predicted average survival (%) of field populations of *S. frugiperda* from different regions of Brazil monitored for three diamide insecticides: flubendiamide, chlorantraniliprole, and cyantraniliprole. Data on the insecticides flubendiamide and chlorantraniliprole were obtained from Ribeiro,^38^ Bolzan,^37^ and Okuma *et al*.^23^

When comparing cyantraniliprole with other diamides, such as flubendiamide and chlorantraniliprole, which were released in mid‐2010, survival rates for these older diamides remained below 10% during the first 4 years of monitoring (2014–2018), except for BA (Fig. 4). In regions with intensive agricultural production systems, including the Brazilian Cerrado States (BA, MT, GO, MS, and MG), there was a slight increase in survival rates. Field populations from southern Brazil (Paraná‐PR, Santa Catarina‐SC, and Rio Grande do Sul‐RS) consistently showed average survival rates below 10% for all diamides, indicating stable susceptibility.

**Figure 5:**
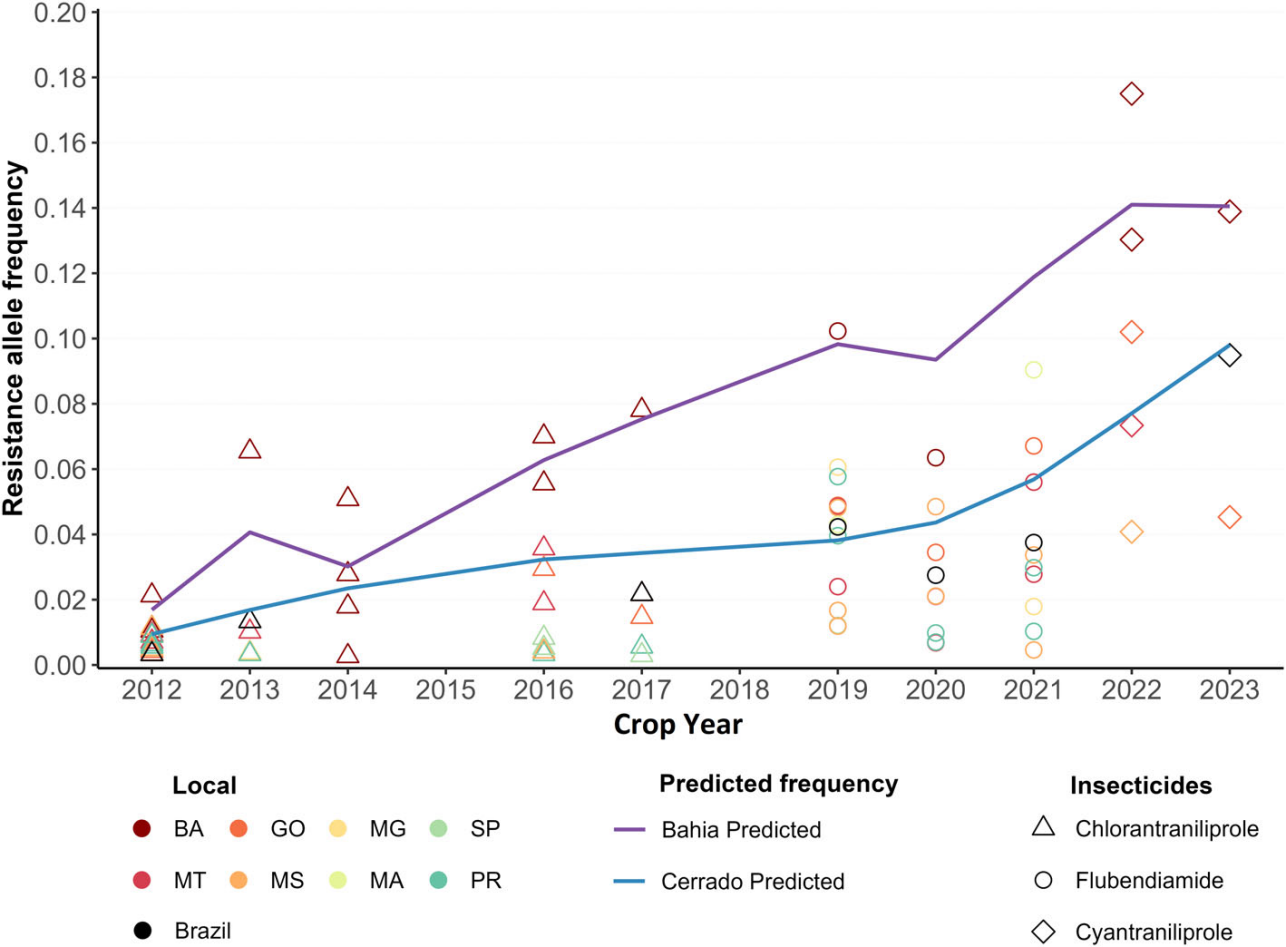
Estimates of the frequency of diamide‐resistant alleles (I4790K and I4790M) throughout the crop seasons in Brazil. The predicted models (solid line) were adjusted using the GLMM with data reported by Ribeiro,^38^ Bolzan,^37^ and Okuma *et al*.,^39^ encompassing resistance frequencies to cyantraniliprole, chlorantraniliprole, and flubendiamide.

An overall increasing trend in the estimated resistance allele frequency was observed across crop seasons in Brazil (Fig. 5). Predictive models adjusted using the GLMM incorporated our data on cyantraniliprole along with information from Ribeiro,^38^ Bolzan,^37^ and Okuma *et al*.,^39^ regarding chlorantraniliprole and flubendiamide indicating a consistent increase in resistance alleles for diamides over time. BA highlighted a significant increase in resistance allele frequency compared to the Cerrado average, indicating stronger selective pressure in this region. This rise in resistance evolution in BA may be due to local agricultural practices (three crop seasons and large arable land) that intensify this pressure and promote the spread of resistant alleles.

### 3.6 Molecular and genotypic analysis of the ryanodine receptor in cyantraniliprole‐resistant *Spodoptera frugiperda*

**Figure 6:**
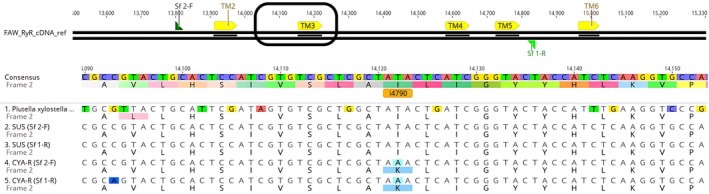
Mutation region between transmembrane domains 2 and 3 (TM2 and TM3) of the ryanodine receptor channel in cyantraniliprole‐resistant *S. frugiperda*. Cyan‐colored ‘A’ letters indicate the base‐pair substitution site in the resistant strain CYA‐R at position 4790 in *P. xylostella* (corresponding to I4734K in *S. frugiperda*).

**Figure 7:**
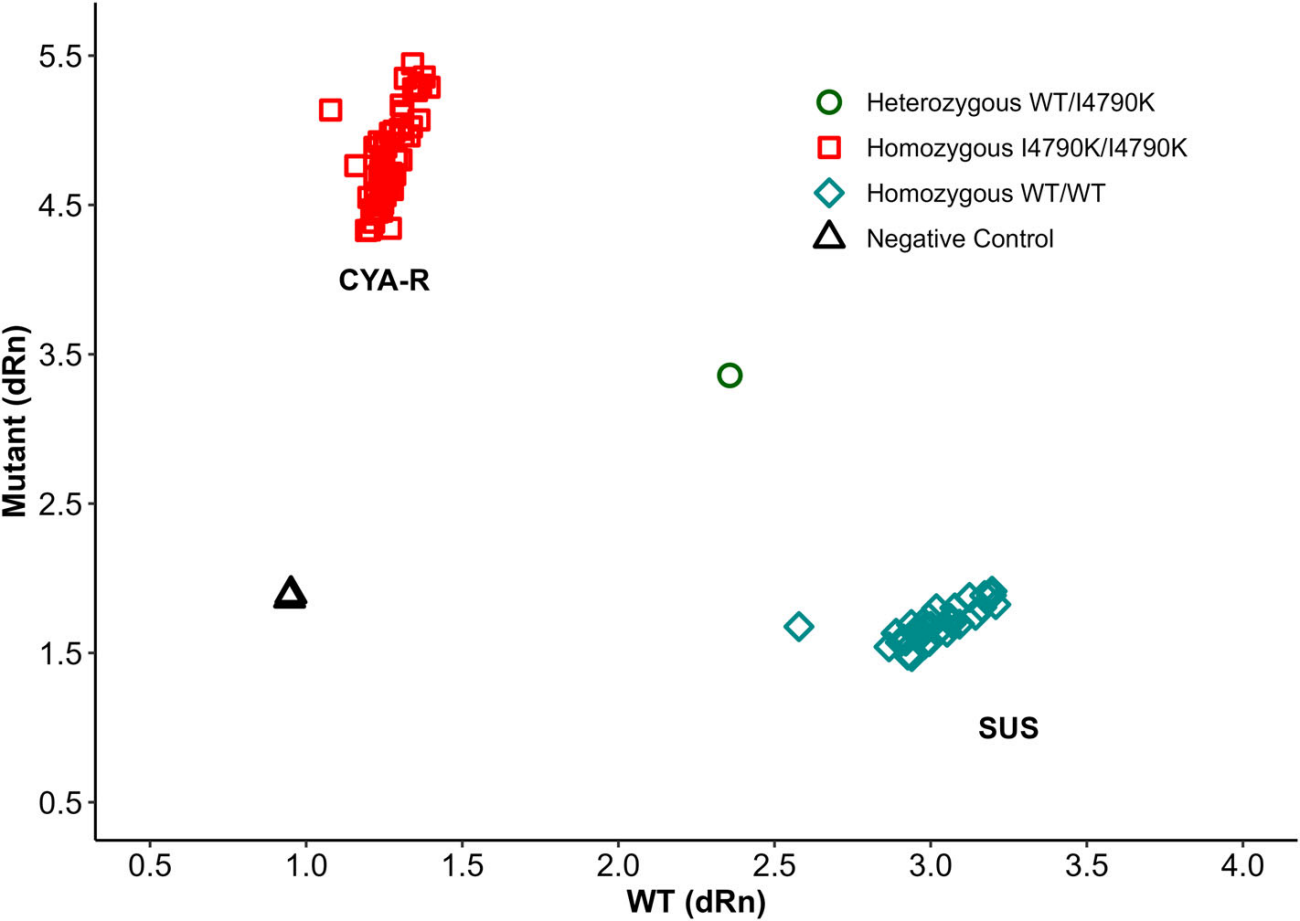
Allelic discrimination plot of the normalized reporter signal from the allele 1 probe plotted against the normalized reporter signal from the allele two probe from *S. frugiperda* larvae resistant and susceptible to cyantraniliprole for the I4790K mutation.

Sequencing the transmembrane domain of the ryanodine receptor in CYA‐R strain revealed substitution of thymine (T) by adenosine (A), leading to an aminoacidic lysine (K) replacing isoleucine (I) at position 4734 in *S. frugiperda*, corresponding to the I4790K mutation in *P*. *xylostella* (NP_001296002) (Fig. 6). Genotyping of 30 larvae isolated from the cyantraniliprole‐resistant strain (CYA‐R) showed that 100% of the individuals were homozygous for the I4790K mutation (Fig. 7). Since most resistance‐associated mutations in the ryanodine receptor have been described in this transmembrane region, our results suggest that the I4790K mutation plays a role in cyantraniliprole resistance. However, other potential mechanisms cannot be ruled out as they were not explored in this study.

## 4 DISCUSSION

The monitoring results indicated trends in the survival rates of *S. frugiperda* using cyantraniliprole. Field populations in regions characterized by intensive agricultural practices, particularly within the Brazilian Cerrado (including Bahia‐BA, Mato Grosso‐MT, Goiás‐GO, Minas Gerais‐MG, and Mato Grosso do Sul‐MS), demonstrated significantly increased survival rates within the crop years. While the average survival rates remained relatively stable across most regions, the increase in resistance frequencies in these areas indicates a clear need for effective management strategies to address this issue.

In contrast, in southern Brazil, where agricultural production systems and climatic conditions differ markedly from the Cerrado, *S. frugiperda* appears to exhibit less adaptation to these environments.^51^, ^52^ Thus, these conditions in southern Brazil, enable the strategic use of diamides with integrated pest management practices, including rotation of insecticides with different modes of action. Notably, baculovirus SfMNPV and sodium channel blockers exhibit low susceptibility variability among *S. frugiperda* populations in Brazil, with resistance ratios below 4.06‐fold^53^ and 4.6‐fold,^54^ respectively, being two interesting options for an integrated pest management approach.

Diamides are commonly recommended for seed treatment because of their favorable insecticidal properties, such as residual activity and translocation within plants.^9^ However, repeated applications of diamides in regions with intensive production systems, like BA, MT, and GO, could accelerate the development of resistance. The monitoring results suggest that resistance management strategies should be prioritized in these regions, as we observed reduced susceptibility to chlorantraniliprole and flubendiamide and a recent trend of higher survival rates for cyantraniliprole since the 2022 crop season.

Initial studies in 2014 reported low resistance frequencies for diamides in *S. frugiperda*, with values ranging from 0.0033 to 0.0125 for chlorantraniliprole in Brazil.^38^ However, studies conducted in 2019 have shown that the frequency of chlorantraniliprole was 0.0781.^37^ In our study, we observed a higher resistance allele frequency of 0.1442 for cyantraniliprole in western BA, a region in which we also selected a resistant strain. Previous investigations have identified the heterozygous I4790M/K mutation in *S. frugiperda* individuals selected for flubendiamide, demonstrating high cross‐resistance to cyantraniliprole.^23^ However, our study is the first to report a *S. frugiperda* strain selected with cyantraniliprole that is entirely homozygous for the I4790K mutation, which represents one of the possible resistance mechanisms. The detailed characterization of this mutation provides clear evidence of its complete involvement in the insect's biological response, leading to reduced susceptibility to diamide insecticides, including chlorantraniliprole, cyantraniliprole, cyclaniliprole and flubendiamide. Consequently, alleles associated with diamide resistance are widespread in the field, emphasizing the necessity to monitor the survival of field populations and estimate the frequency of resistance alleles to establish an effective resistance management program.

A key finding of our study was the successful selection of a cyantraniliprole‐resistant strain of *S*. *frugiperda* (CYA‐R) by F_2_ screen, which exhibited high levels of cross‐resistance to all diamides tested. Thus, the rapid increase in resistance frequency may be driven by cross‐resistance between diamides, where individuals resistant to one diamide are also unaffected by others, accelerating the spread of resistance alleles in the field. The overlap of confidence intervals among heterozygous strains resulting from reciprocal crosses between susceptible and resistant strains of *S. frugiperda* to cyantraniliprole indicates an autosomal inheritance pattern. This finding suggests that the traits associated with resistance are located on autosomal chromosomes, excluding the possibility of maternal effects or sex‐linked inheritance. Additionally, this observation is consistent with previous studies on diamide resistance in *S. frugiperda* strains resistant to chlorantraniliprole and flubendiamide.^23^, ^55^ The recessive nature of resistance is promising, as heterozygotes behave similarly to susceptible individuals, which may help delay the evolution of resistance.^56^, ^57^, ^58^ Resistance increases more slowly when resistance inheritance is recessive because the alleles that confer resistance are rare, and the resistant phenotype only manifests in homozygotes.^59^, ^60^

The selection pressure applied to the CYA‐R strain during the study was approximately 3200 μg a.i. mL^−1^ of cyantraniliprole, which was 20‐times the LC_99_ for heterozygotes. This high selection pressure ensured the strain was fully homozygous for the I4790K mutation, significantly reducing the likelihood of susceptible/heterozygous individuals contaminating the strain. However, our results demonstrated that even commercially pure products of other diamides, such as chlorantraniliprole and flubendiamide, could not achieve 50% mortality in the CYA‐R strain. These findings indicate that cyantraniliprole‐resistant *S. frugiperda* populations exhibit significant cross‐resistance to other diamides, posing a considerable challenge to Integrated Pest Management (IPM) programs relying on these insecticides. To delay the evolution of resistance, it is essential to implement Insecticide Resistance Management (IRM) strategies within IPM programs, particularly through the rotation of chemical groups to prevent high selection pressure on field populations,^61^, ^62^, ^63^ especially in cases where individuals display cross‐resistance, as observed in our study.

We hypothesized that the observed increase in survival among field populations over the past 2 years is due to the rising prevalence of mutations in the 4790 amino acids of the ryanodine receptor (I/M, I/K), as initially reported by Okuma *et al*.^23^ These researchers found a predominance of susceptible individuals (90% I/I) in field populations, followed by heterozygotes for the I/M mutation (<7%), rare occurrences of resistant homozygotes (M/M) (<2%), and less than 1% of heterozygotes with the I4790K mutation (I/K). Despite the low frequency of I/K heterozygotes, we successfully selected a 100% homozygous strain for the I4790K mutation using the F_2_ screening method in our study. This suggests that the frequency of individuals with this mutation may increase in the field. Therefore, monitoring programs are essential to track the frequency of these mutations, particularly I4790K, as they can provide critical information for resistance management.

Monitoring studies such as those reported by Okuma *et al*.,^23^ as well as those conducted in China,^29^ revealed a rapid increase in the proportion of individuals with the I/K mutation, leading to high resistance rates to chlorantraniliprole in *P. xylostella*. This emphasizes the need for monitoring strategies to delay the evolution of resistance by implementing IRM practices. Early detection of resistance alleles allows for proactive adjustments in control strategies, such as implementing insecticide rotations before resistance becomes widespread. Additionally, monitoring can identify populations where the resistant allele is still at a low frequency, enabling targeted interventions to slow their spread and preserve the effectiveness of insecticides like cyantraniliprole. By integrating monitoring data with IRM and IPM strategies, it is possible to mitigate selection pressure and extend the lifespan of available insecticides.

The introduction of the I4790K mutation into *P. xylostella* has been shown to confer significant cross‐resistance to all diamides, making this mutation particularly relevant in this species.^29^ Molecular docking studies revealed that the mutation disrupts key hydrogen bonds within the ryanodine receptor's binding pocket, significantly reducing binding affinity.^29^ Similarly, Zhao *et al*.^64^ found that, despite the mutation, cyantraniliprole retains high activity through additional van der Waals interactions that compensate for the loss of hydrogen bonds, whereas flubendiamide exhibits markedly reduced binding, consistent with its extremely high resistance ratio. Du & Fu^65^ further emphasized that subtle chemical differences among diamides lead to distinct binding modes and resistance profiles. Collectively, these findings underscore that while the I4790K mutation is pivotal in mediating resistance, the inherent structural features of each diamide insecticide also critically influence their binding efficacy and resistance phenotypes. Although the role of the I4790K mutation in *S. frugiperda* has not been as extensively documented, our findings raise serious concerns about resistant fall armyworm populations carrying the I4790K mutation. This issue is particularly alarming, as individuals with this mutation show a lack of responsiveness to the currently available diamides.

In future studies, we will explore the potential fitness cost associated with the CYA‐R strain, which was selected in the current research and comprised 100% of the individuals carrying the I4790K mutation. We aim to assess whether this mutation confers disadvantages on resistant *S. frugiperda* individuals. Previous laboratory investigations in *P. xylostella* have indicated a relatively low fitness cost for this mutation compared to a susceptible laboratory strain (R_0_ = 0.92).^34^ The findings raise concerns about the increased adaptability of resistant individuals, which may contribute to the evolution of resistance in field populations.

## 5 CONCLUSION

Our study provides valuable insights into the mechanisms of *S*. *frugiperda* resistance to cyantraniliprole. We focused on selecting a resistant strain (CYA‐R) that is both homozygous and functionally recessive, demonstrating high resistance to cyantraniliprole and other diamides. The I4790K mutation plays a crucial role in resistance to cyantraniliprole as well as in cross‐resistance to chlorantraniliprole, cyclaniliprole and flubendiamide. It contributed to understanding the increased resistance frequency observed in regions such as the Cerrado, particularly in Bahia (BA), where intensive agricultural practices are widespread. The selection of this resistant strain highlights the potential for cross‐resistance to accelerate the spread of resistance alleles within field populations. To effectively address this challenge, resistance management strategies should focus on monitoring programs, resistant allele detection and quantification, and the rotation of insecticides with different modes of action to mitigate further resistance development.

## CONFLICT OF INTEREST

Jackeline P Borba and Eduardo CM Picelli are employed by FMC Agricultural Solutions. This research was partially financed by FMC Agricultural Solutions. These authors declare no additional conflict of interest. Leonardo V Thiesen, Gabriela C Gonçalves, Aline S Guidolin, Antonio RB Nascimento, Everton F Coutinho, and Celso Omoto declare no potential conflict of interest.

## Supporting information


**Table S1.** Survival (%), collection location, and sample size (*n*) of *Spodoptera frugiperda* field populations screened for resistance monitoring using the diagnostic dose (180 μg a.i. of cyantraniliprole) from 2017 to 2023 crop seasons.

## Data Availability

The data that support the findings of this study are available from the corresponding author upon reasonable request.
